# Relationships between Signaling Pathway Usage and Sensitivity to a Pathway Inhibitor: Examination of Trametinib Responses in Cultured Breast Cancer Lines

**DOI:** 10.1371/journal.pone.0105792

**Published:** 2014-08-29

**Authors:** Euphemia Y. Leung, Ji Eun Kim, Marjan Askarian-Amiri, Gordon W. Rewcastle, Graeme J. Finlay, Bruce C. Baguley

**Affiliations:** Auckland Cancer Society Research Centre, University of Auckland, Auckland, New Zealand; Taipei Medical University, Taiwan

## Abstract

Cellular signaling pathways involving mTOR, PI3K and ERK have dominated recent studies of breast cancer biology, and inhibitors of these pathways have formed a focus of numerous clinical trials. We have chosen trametinib, a drug targeting MEK in the ERK pathway, to address two questions. Firstly, does inhibition of a signaling pathway, as measured by protein phosphorylation, predict the antiproliferative activity of trametinib? Secondly, do inhibitors of the mTOR and PI3K pathways synergize with trametinib in their effects on cell proliferation? A panel of 30 human breast cancer cell lines was chosen to include lines that could be classified according to whether they were ER and PR positive, HER2 over-expressing, and “triple negative”. Everolimus (targeting mTOR), NVP-BEZ235 and GSK2126458 (both targeting PI3K/mTOR) were chosen for combination experiments. Inhibition of cell proliferation was measured by IC_50_ values and pathway utilization was measured by phosphorylation of signaling kinases. Overall, no correlation was found between trametinib IC_50_ values and inhibition of ERK signaling. Inhibition of ERK phosphorylation was observed at trametinib concentrations not affecting proliferation, and sensitivity of cell proliferation to trametinib was found in cell lines with low ERK phosphorylation. Evidence was found for synergy between trametinib and either everolimus, NVP-BEZ235 or GSK2126458, but this was cell line specific. The results have implications for the clinical application of PI3K/mTOR and MEK inhibitors.

## Introduction

The MAPK (Mitogen activated protein kinase) pathway (RAS-RAF-MEK-ERK) and PI3K-AKT-mTOR pathways play dominant roles in regulating diverse cellular processes, including proliferation and survival, in breast cancer. These pathways have been identified as important for breast cancer behavior for a number of years [1], [2] and interact strongly with the estrogen receptor (ER) pathway, as shown by cross-talk in the development of tamoxifen resistance in breast cancer [3], [4]. Increased EGFR signaling through the MAPK pathway occurs frequently both clinically and in cancer cell lines that have developed resistance to endocrine therapies [5], [6]. In addition, activation of the MAPK pathway is associated with increased risk of metastasis [7]. As signaling networks integrate multiple upstream inputs, inhibition of MEK is an attractive cancer therapeutic strategy [1]. Although the MAPK pathway is a validated therapeutic target in breast cancer, the mechanisms underlying the poor clinical response to MEK inhibition remain unclear. Tumors with RAS/RAF mutations seem to be more sensitive to MEK inhibitors but their responses are not uniform [8]. Activating mutations in PIK3CA, affecting the PI3K-AKT-mTOR pathway, are frequent in breast cancer [9] and raise the question of whether they alter the balance of pathway utilization.

Since MEK is the downstream effector of BRAF, MEK inhibition is an attractive strategy to block activation of the MAPK pathway and could also potentially block reactivation of the MAPK pathway in BRAF inhibitor–resistant disease [10]. In a small number of melanoma lines, the pattern of ERK (MEK effector) phosphorylation inhibition broadly followed that of the IC50 results [11]. However, MEK inhibitors have shown minimal clinical activity in tumors with activating BRAF mutations, as observed with sequential therapy in patients previously treated with a BRAF inhibitor, suggesting that BRAF-inhibitor resistance mechanisms likely confer resistance to MEK-inhibitor monotherapy [12]. Triple negative breast cancer cell lines were shown to be more sensitive to trametinib than cell lines from other breast cancer subtypes [13]. Trametinib (GSK1120212) is a potent and specific MEK1/2 allosteric inhibitor that is under clinical study to define the kinase response in triple negative breast cancer (NCT01467310). It has been recently approved for treating unresectable or metastatic melanoma with BRAF ^V600E^ or ^V600K^ mutations [14].

**Figure 1:**
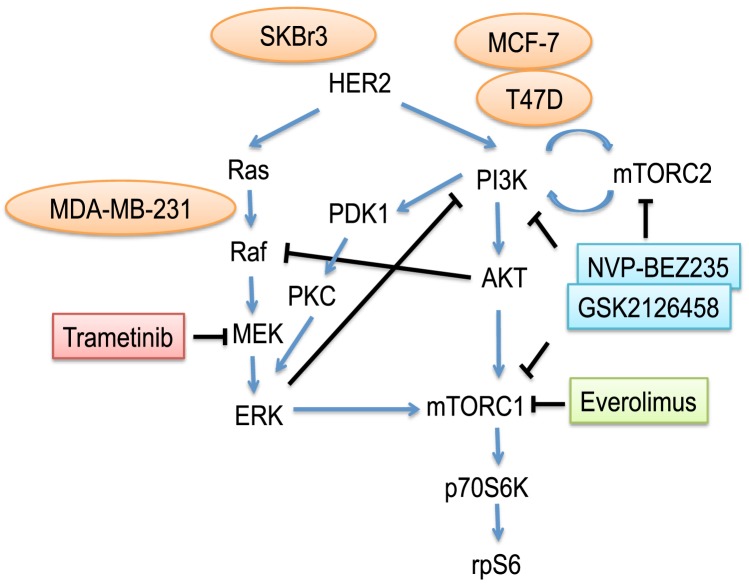
Schematic representation of a network of PI3K, mTOR and MEK complex signaling. Blue arrows and black lines represent activating and inhibitory connections, respectively.

We have chosen trametinib [15] to address the following question with respect to the behavior of breast cancer cell lines: does inhibition of a signaling pathway, as measured by suppression of protein phosphorylation, predict the antiproliferative activity of a pathway inhibitor? We have used the inhibitors everolimus (mTOR) [16], NVP-BEZ235 and GSK2126458 (PI3K/mTOR) [17]–[19] to test for possible pathway interactions with trametinib (Figure 1). Initially, we selected four breast cancer cell lines: MCF-7 and T47D (ER+, mutant PIK3CA E545K and H1047R, respectively), SKBr3 (HER2+) and MDA-MB-231 (triple negative/basal B, mutant KRAS G13D, BRAF G464V) [20], to determine whether firstly the sensitivities to the MAPK pathway inhibitor trametinib correlate with the activity of the corresponding pathway. We then extend our study with a panel of 30 breast cancer cell lines to confirm our initial finding.

Recently, co-targeting the PI3K and RAS pathways was shown to be an effective therapy *in vivo* compared with single agents alone in neuroendocrine tumors [21]. The combinations of MEK and PI3K/mTOR inhibitors can induce synergistic growth inhibition [22]. Clinical studies using PI3K/mTOR inhibitors in combination with MEK inhibitor (NCT01347866, NCT01337765, NCT01390818) are under investigation. The published data suggest that the combined effects of the PI3K/mTOR and MEK pathways may be important for cancer growth, pointing to the possibility that combined blockage of these two signaling pathways may be an important component of successful breast cancer treatment strategies. In this study, we have utilized the Chou-Talalay method [23] to examine the question of whether inhibitors of the mTOR and PI3K pathways synergize with ERK pathway inhibitors in their effects on breast cancer cell lines

## Material and Methods

### Cell culture

Culture conditions have been described previously [24]; MCF-7, T47D, SKBr3, MDA-MB-468, BT20, MDA-MB-231, HCC1143, HCC70, SUM149PT, HCC1954, MDA-MB-436, HPL100, BT549 and SUM159PT cells were purchased from the American Type Culture Collection (ATCC). PMC42ET [25] was a kind gift from Dr. Chanel Smart (The University of Queensland). SUM149PT and SUM150PT were grown in Ham's F-12 with 5% fetal bovine serum (FBS) supplemented with 5 µg/mL insulin, 1 µg/mL hydrocortisone, 10 mM HEPES and penicillin/streptomycin (100 U/ml and 100 µg/ml, respectively). Apart from MCF-7 sub-lines, all cells were grown in α-MEM containing 5% FBS. All the other growth media contained insulin/transferrin/selenium supplement, added according to the manufacturer’s instructions (Roche), as well as penicillin/streptomycin (100 U/ml and 100 µg/ml, respectively). The TamR7 cell line was established by culturing MCF-7 cells in the presence of progressively increasing concentrations of tamoxifen (0.1 – 3 µM; stock dissolved in DMSO) and then maintaining them for >15 months in 3 µM tamoxifen. The TamR3 and TamR6 cell lines were generated by growth of MCF-7 cells in phenol-red-free RPMI containing 10% charcoal-stripped fetal bovine serum (Invitrogen, Auckland, NZ), over a period of 3 months to progressively increasing concentrations of tamoxifen (1 nM to 1 µM in ethanol) and then maintaining them for >15 months in 1 µM tamoxifen. The TamC3 and TamC6 cell lines were generated by exposure of MCF-7 cells for >16 months to the above growth medium but lacking tamoxifen [24], [26], [27]. The FulvR1a, FulvR1c and FulvR2a cell lines were generated by growth of MCF-7 cells in phenol red-free RPMI containing 5% charcoal-stripped fetal bovine serum, over a period of 3 months in progressively increasing concentrations of fulvestrant (1 nM to 100 nM; stock dissolved in ethanol), and then maintaining them for >12 months in 100 nM fulvestrant. The FulvC1a, FulvC1b and FulvC2 cell lines were generated by exposure of MCF-7 cells for >12 months to the above growth medium but lacking fulvestrant [28]. All experiments were carried out in cells grown in their respective growth media but without tamoxifen or fulvestrant.

### Chemicals and reagents

Tamoxifen was purchased from Sigma (Auckland, NZ). Everolimus and trametinib were purchased from Selleck Chemicals (Houston, USA). Trametinib was also obtained from Jinan Trio Pharmatech co., Ltd (Jinan, China). NVP-BEZ235 [29], [30] and GSK2126458 [17] were synthesized according to published protocols.

### Cell proliferation assay

Cell proliferation was measured using thymidine incorporation in which 3000 cells were seeded in 96 well plates in the presence of varying concentrations of inhibitors for 3 days. Briefly, 0.04 µCi of ^3^H-thymidine was added to each well and incubated for 6 h, after which the cells were harvested onto glass fiber filters using an automated TomTec harvester. Filters were incubated with Betaplate Scint and thymidine incorporation measured in a Trilux/Betaplate counter. Effects of inhibitors on cell proliferation were determined relative to the incorporation of ^3^H-thymidine into DNA of control (non-drug-treated) cells.

### Western blotting

Breast cancer cell lines were grown to log-phase, washed twice with ice-cold PBS, and lysed in SDS lysis buffer according to the manufacturer’s protocol (Cell Signaling Technology, Danvers, MA). Protein concentration was quantified using the bicinchoninic acid reagent (Sigma). Cell lysates containing 25 µg of protein were separated by SDS-PAGE gel electrophoresis, and transferred to PVDF membranes (Millipore). Membranes were immunoblotted with antibodies against phospho-Akt (S473), total Akt, phospho-p70S6K (T389), total p70S6K, phospho-rpS6 (S235/236), total rpS6, phospho-ERK (T202/Y204), total ERK, (all from Cell Signaling Technology), tubulin (Sigma), and actin (Millipore). Bound antibody was visualized using SuperSignal West Pico (Thermo Scientific, Waltham, MA) or ECL plus (GE Healthcare, Auckland, NZ) and the chemiluminescence detection system by Fujifilm Las-3000. Densitometry was performed using ImageJ. The relative intensity of phosphorylated proteins was normalized using either tubulin or actin as the standard. The fold change was then calculated between MCF7 and BT20 as standard reference.

### Data analysis

The Chou-Talalay method for drug combination is based on the median-effect equation and was used to indicate drug interactions [23]. The resulting combination index (CI) theorem of Chou-Talalay offers quantitative definition for synergism, additivity and antagonism, where simplified CI values and their indication are define as synergism (CI<0.9), additive effect (0.90≤CI≤1.1), and antagonism (1.1≤CI≤10) in drug combinations. The CompuSyn software (http://www.combosyn.com/) was used to calculate combination index.

Data were analyzed using a one-way ANOVA coupled with multiple comparisons versus treatment control applying the Holm-Sidak method correction, where *p*<0.05 denotes a statistically significant difference. T-test or Mann-Whitney Rank Sum Test was used for comparison between two groups. Correlation analysis was performed with Spearman’s rank correlation coefficient (*R)* and statistical significance (*P)* using SigmaPlot. Values of *P*<0.05 were considered to be statistically significant.

## Results

### Relationship between trametinib-induced growth inhibition and ERK phosphorylation in MCF-7, T47D, SKBr3 and MDA-MB-231 breast cancer cell lines

**Figure 2:**
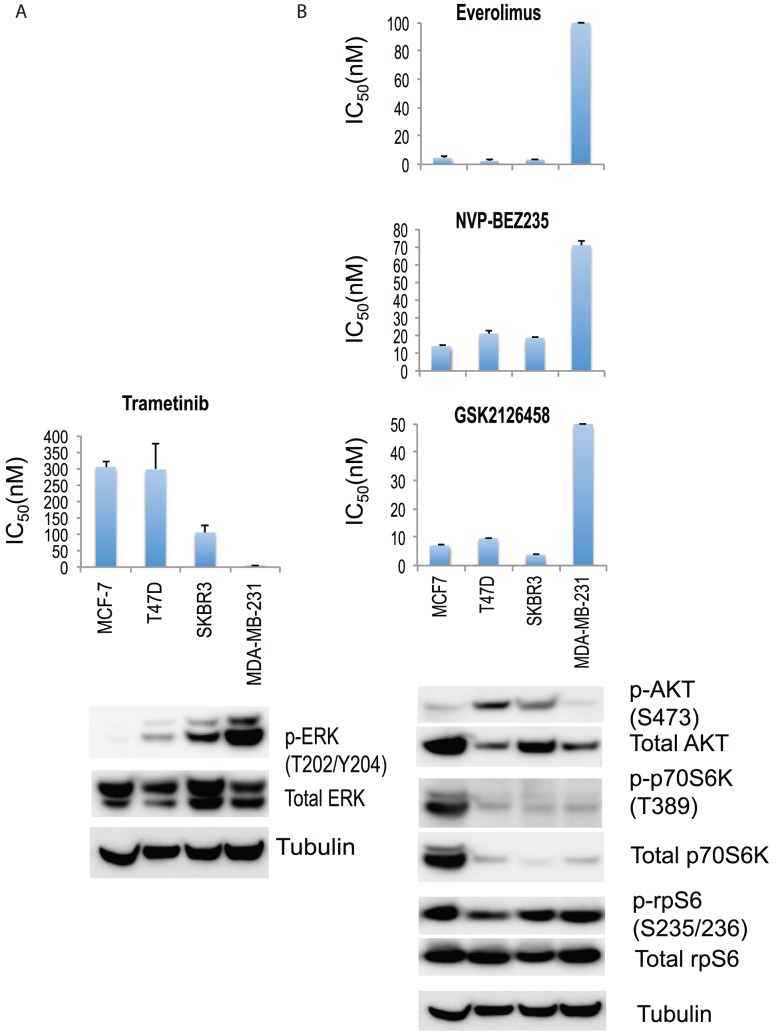
Growth inhibitory concentrations for MCF-7, T47D, SKBr3 and MDA-MB-231 exposed to different drugs. IC50 values (50% inhibition of growth) are shown for (A) trametinib, as well as (B) everolimus, NVP-BEZ235, and GSK2126458. Cells were treated with drugs for 3 days and cell proliferation was measured by the thymidine incorporation assay. Results are shown as the mean *±* standard error from triplicate experiments. Phosphorylation of (A) ERK, and (B) AKT, p70S6K and S6 in MCF-7, T47D, SKBr3 and MDA-MB-231 cell lines. Immunoblots with antibodies specific for phosphorylated proteins and their respective total protein are indicated below. Tubulin is the loading control.

**Figure 3:**
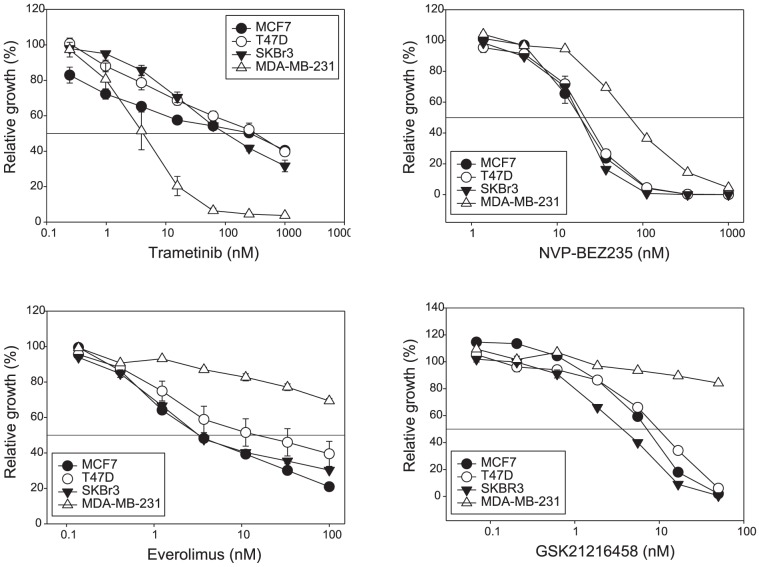
Growth inhibitory effects of drug treatments in MCF-7, T47D, SKBr3 and MDA-MB-231 cell line. Cells were treated with trametinib, everolimus, NVP-BEZ235 or GSK2126458 for 3 days and [^3^H]-thymidine added for the last 6 hours. Results are shown as the mean *±* standard error from duplicate experiments.

We initially chose four breast cancer cell lines characterised by a diversity of activated signaling pathway and estrogen receptor (ER) expression: MCF-7 and T47D were ER+, SKBr3 was HER2+ and MDA-MB-231 was triple-negative. PI3K and MAPK pathway usage was analyzed by western blots (Figure 2A and B). The cell lines were assayed for sensitivity to trametinib, using a 3-day ^3^H-thymidine incorporation proliferation assay. Results were compared to those of everolimus, NVP-BEZ235 and GSK2126458 (Figure 2 and 3). MDA-MB-231, with mutant RAS and RAF proteins, showed high levels of phosphorylated ERK (p-ERK) expression indicating active MAPK pathway signaling, and also high sensitivity to the MEK inhibitor trametinib (Figure 2A). Lower levels of p-ERK expression were observed in SKBr3 and T47D (mutant PIK3CA H1047R), and MCF-7 (mutant PIK3CA E545K) showed minimal basal p-ERK expression. In the MCF-7 line, the gene encoding p70S6K was amplified, with corresponding overexpression of protein [31] and p70S6K phosphorylation; it also showed high sensitivity to the mTOR inhibitor everolimus, and to the PI3K/mTOR inhibitors NVP-BEZ235 and GSK2126458 (Figure 2B). For these four cell lines, the level of protein phosphorylation in the PI3K/mTOR and MAPK pathways appeared to be related to sensitivity to the corresponding inhibitors.

**Figure 4:**
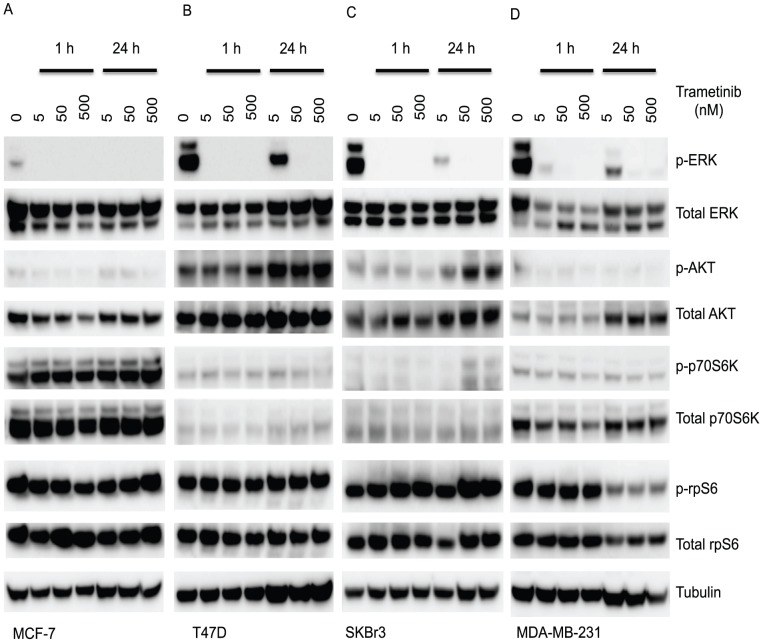
Trametinib effect on phosphorylation of ERK, AKT, p70S6K and rpS6 in breast cancer cell lines. Phosphorylation of ERK, AKT, p70S6K and rpS6 in (A) MCF-7, (B) T47D, (C) SKBr3 and (D) MDA-MB-231 cell lines treated with indicated concentration of trametinib for 1 or 24 h. Immunoblots for phospho-proteins and their respective total proteins are indicated below. Tubulin is the loading control.

The cellular response of the cell lines to trametinib was then assessed by measuring phosphorylation of ERK (Figure 4). Phosphorylation was measured both 1 h and 24 h after addition of drug. For the triple-negative MDA-MB-231 line, trametinib concentrations that inhibited ERK phosphorylation also inhibited proliferation. Unexpectedly, however, trametinib inhibited ERK phosphorylation (p-ERK) in the MCF-7, T47D and SKBr3 cell lines at concentrations with little or no effect on proliferation (Figure 2A). This result showed in these cell lines that the level of p-ERK was markedly reduced, as early as 1 h, and at concentrations of 50 nM and 500 nM, at which p-ERK remained undetectable after 24 h. The loss of ERK phosphorylation (Figure 4A, B, C and D) showed no relationship to the degree to which proliferation was inhibited by trametinib (Figure 2A).

### Effects on proliferation of combinations of trametinib with mTOR/PI3K inhibitors

**Figure 5:**
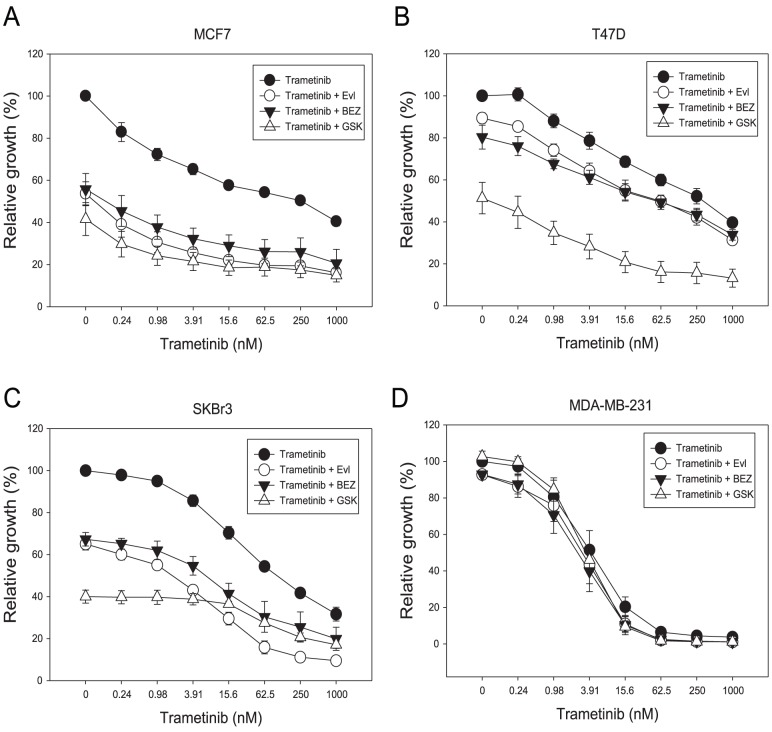
Growth inhibitory effects of combined drug treatments in MCF-7, T47D, SKBr3 and MDA-MB-231 cell lines. Cells were treated with trametinib in association with everolimus, NVP-BEZ235 or GSK2126458 for 3 days with [^3^H]-thymidine added for the last 6 hours. Results were shown as the mean ± standard error from duplicate experiments.

**Figure 6:**
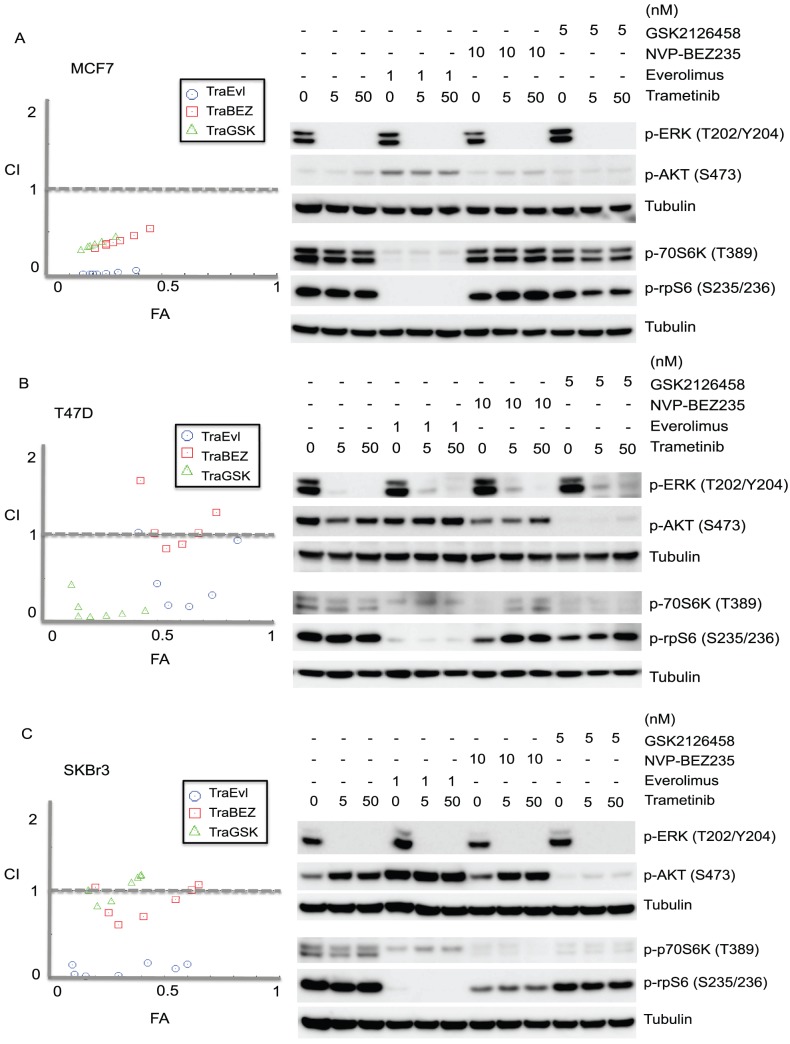
The effects of drug combinations on MCF-7, T47D and SKBr3 cell lines. Left hand panels: Growth inhibitory effects of combinations of trametinib with everolimus (TraEvl), NVP-BEZ235 (TraBEZ) and GSK2126458 (TraGSK) using the Chou-Talalay method. The combination index (CI) theorem of Chou-Talalay defines Synergism: CI<0.9; additivity: 0.90≤CI≤1.1; antagonism: 1.1≤CI≤10 in drug combinations. X axis, FA, the ratio of the fraction affected; y axis, CI, the combination index. Results were from two independent experiments. Right hand panel: Drug combination effect on phosphorylation of ERK, AKT, p70S6K and rpS6 in (A) MCF-7, (B) T47D, (C) SKBr3 cell lines after 24 h as demonstrated by immunoblotting with anti-phospho antibodies. Tubulin is the loading control.

Although trametinib did not inhibit proliferation of the MCF-7, T47D and SKBr3 lines at the concentrations at which it fully suppressed ERK phosphorylation, it was possible that its effect on ERK phosphorylation could sensitize these cells to inhibition by other drugs. We therefore measured the effects of combinations of trametinib and PI3K/mTOR inhibitors (everolimus, NVP-BEZ235, or GSK2126458) (Figure 5 and 6). The Chou-Talalay combination index (CI) for assessing effects of multiple drug exposures was used to indicate drug interactions [23]. The average CI values for combinations of trametinib with everolimus were <0.9, indicating synergy for MCF-7, T47D, SKBr3 and MDA-MB-231 cells (Table 1 and Figure 5). However, combination of trametinib and NVP-BEZ235 in T47D cells were 1.1, suggesting additive effect (Table 1). This result suggested that trametinib, at concentrations that did not inhibit proliferation by itself, increased the growth inhibitory effects of drugs acting on other signaling pathways.

**Table 1:** Combination index for the combination of trametinib and PI3K/mTOR inhibitors in three cell lines tested.

		Combination index			
	CI	MCF-7	T47D	SKBr3	MDA-MB-231
Everolimus	Range	0.01 – 0.06	0.19 – 1.03	0.03 – 0.18	0.09 – 0.69
	Average	0.03±0.02	0.52±0.37	0.10±0.07	0.39±0.24
NVP-BEZ235	Range	0.32 – 0.54	0.84 – 1.62	0.61 – 1.07	0.20 – 1.15
	Average	0.40±0.08	1.11±0.29	0.87±0.18	0.53±0.34
GSK2126458	Range	0.30 – 0.45	0.07 – 0.44	0.83 – 1.18	0.09 – 1.52
	Average	0.36±0.05	0.16±0.13	1.05±0.15	0.52±5.3

Synergism: CI<0.9; additive: 0.90≤CI≤1.1; antagonism: 1.1≤CI≤10.

Since no significant changes in growth inhibition with concurrent inhibitors treatment were observed in MDA-MB-231 cells (Figure 5), drug effects on the activity of MAPK and PI3K signaling pathways were assessed in the remaining three cell lines: MCF-7, T47D and SKBr3 (Figure 6). Everolimus (1 nM) with or without trametinib was observed to up-regulate p-AKT and markedly reduce p-rpS6 in MCF-7 and SKBr3 cells (Figure 6A and C), and reduce p-p70S6K in MCF-7 cells (Figure 6A). For NVP-BEZ235, increased p-AKT was observed in SKBr3 cells alone or in combination with trametinib, while GSK2126458, with or without trametinib, reduced p-AKT in the T47D and SKBr3 cell lines. The degree to which thymidine incorporation was suppressed in the cell lines tested showed no correlation with the signaling responses (as measured by protein phosphorylation) induced by inhibitors either alone or in combination. We further examined the cell lines for cleaved PARP, a well-characterized marker of apoptosis. No cleaved PARP was detected in any of the cell lines following exposure to trametinib alone or in combination with everolimus, NVP-BEZ235 or GSK2126458 treatment for 24 h (Figure S1).

### Sensitivity of cell proliferation to trametinib in a larger panel of breast cancer cell lines

**Figure 7:**
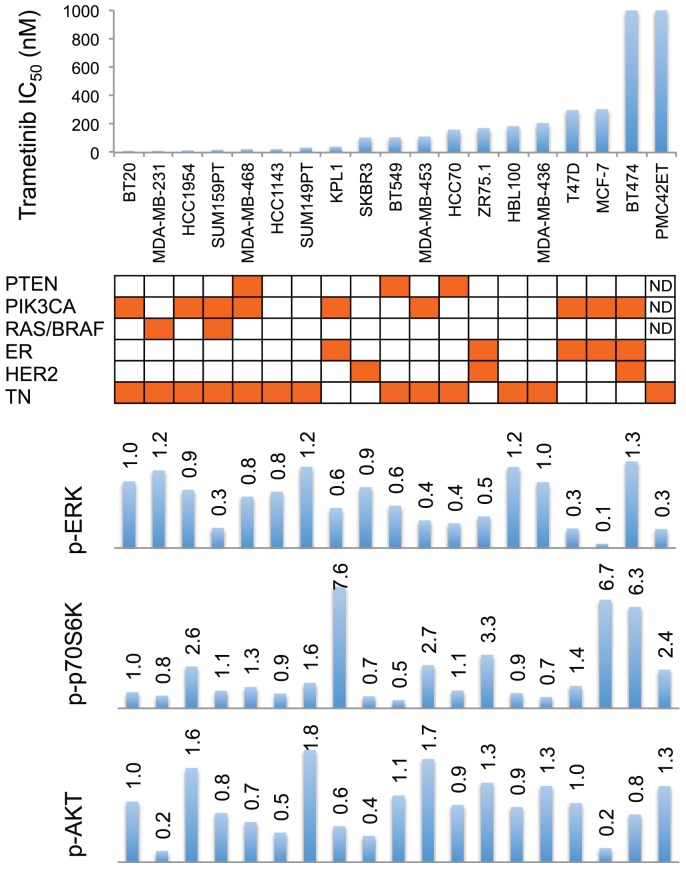
Relationship between drug sensitivity, mutation status, receptor status and pathway utilization. IC_50_ values for trametinib are represented on the y-axis and individual cell lines on the x-axis. Mutations of PTEN, PIK3CA, RAS/BRAF listed. Mutation positive status from The Roche Cancer Genome Database 2.0 [42] are colored orange. Cell lines are colored orange for estrogen receptor positive (ER), HER2 positive and triple-negative (TN) in their respective rows. Relative levels of phosphorylation in p70S6K, AKT and ERK of breast cancer cell lines are shown as bar graphs. Bands are normalized to tubulin control and bars represent changes in fold compared with BT20 and expressed as the mean from two experiments.

The observation (Figure 2) that MDA-MB-231 showed high utilization of the ERK pathway coupled to sensitivity to trametinib raised the question of whether this was generally true for breast cancer lines. We therefore extended this study to a wider variety of lines, as shown in Figure 7 and Figure S2. Contrary to expectation, no significant correlation was observed with signaling pathway utilization (as measured by ERK phosphorylation) and growth repression (as measured by IC_50_ values) by trametinib. However, estrogen receptor positive breast cancer cell lines showed significant resistance (increased IC_50_ values) to trametinib (*p*  = 0.04).

### Sensitivity of cell proliferation to trametinib in a panel of tamoxifen-resistant MCF-7 lines

**Figure 8:**
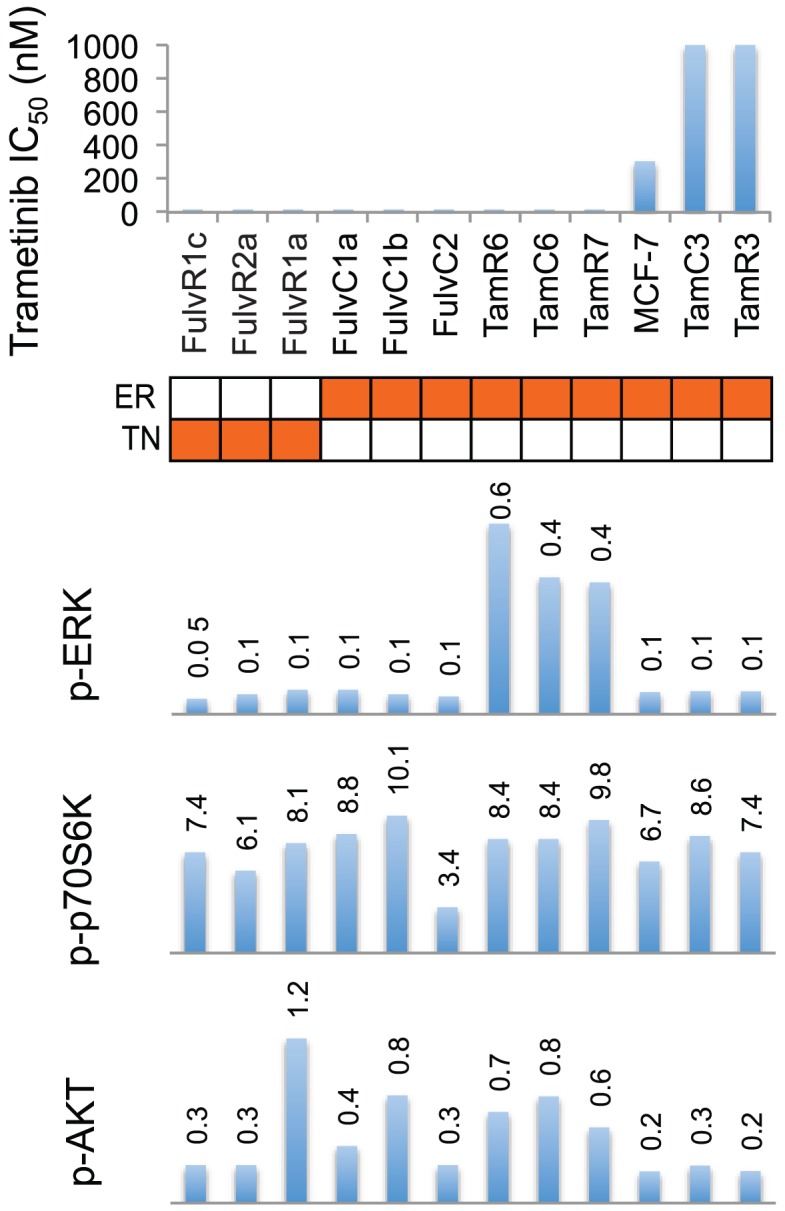
Relationship between drug sensitivity and pathway utilization in MCF-7 parental and sub-lines. IC_50_ values for trametinib are represented on the y-axis and individual cell lines on the x-axis. Relative level of phosphorylation of p70S6K, AKT and ERK of breast cancer cell lines are shown as bar graphs. Bands are normalized to tubulin or actin control and bars represent changes in fold compared with MCF-7 and expressed as the mean from two experiments. Cell lines are colored orange for estrogen receptor positive (ER), and triple-negative (TN) in their respective rows.

The above results referred to cell lines with widely varying properties and we therefore considered whether there would be a relationship between the level of ERK phosphorylation and proliferation inhibition by trametinib in a series of MCF-7 lines that had a common genetic background. Hence, we utilized a series of receptor positive (ER+) MCF-7 tamoxifen-resistant sub-lines [24] and a series of triple negative MCF-7 fulvestrant-resistant sub-lines [28] (Figure 8). Six ER+ lines (TamR7, TamC6, TamR6, FulvC1a, FulvC1b, FulvC2) and three triple-negative (FulvR1a, FulvR1c and FulvR2a) MCF-7 sub-lines showed high sensitivity to trametinib with IC_50_ values of less than 20 nM, while MCF-7 parental (IC50 = 305 nM), TamC3 and TamR3 (both with IC50 > 1000 nM) showed relative resistance to trametinib. No significant correlation was observed between the degree of ERK phosphorylation and trametinib sensitivity (Figure 8).

## Discussion

In this study we have selected a broad series of breast cancer cell lines and investigated their sensitivity to the MEK pathway inhibitor trametinib. As measured by IC_50_ values, trametinib sensitivity varied over a range of more than 180-fold. This range may reflect both the activity of the pathway and differences in drug uptake; trametinib is an inhibitor of ATP-binding cassette efflux transporters such as p-glycoprotein (see MEKINIST Trametinib tablet product monograph), but differences in uptake/efflux should not affect the relationship between sensitivity and pathway utilization. In our series of cell lines, sensitivity did not seem to be related to mutational status (Figures 7 and 8). Triple-negative status also did not stratify cell lines for trametinib sensitivity. This result differs from that of a previous study [13] reporting that cell lines with a basal subtype had increased sensitivity to trametinib, although the authors did state that basal subtype is not equivalent to triple-negative subtype.

We next measured ERK pathway utilization and conclude, for this series of cell lines, that pathway activation cannot predict response to trametinib under standard culture conditions. One possible reason for the lack of correlation is that there are many mechanisms determining trametinib sensitivity or resistance. A recent review has grouped these mechanisms into two categories, ERK dependent and ERK independent [32]. The ERK independent pathways can compensate for the loss of ERK activity, such as when increased signaling from the IGF1R and other RTKs activates AKT signaling where MEK resistant cells displayed elevated levels of AKT phosphorylation [32]. A MEK-independent compensatory ERK activation circuit may be mediated by TOPK or some other unidentified kinase in T47D cells [33], and MEK-independent ERK activation was resistant to inhibition by a number of MEK inhibitors. Resistant hepatocellular carcinoma cells showed a short-term decrease in p-ERK that rebounded to baseline by 24 h, and this suggested that the status of the MEK/ERK pathway in patients could be predictive for response to MEK inhibitors [34]. However, trametinib is a very potent MEK inhibitor and no compensatory ERK activation for up to 24 h was observed in T47D, MCF-7 or SKBr3 cells in our study (Figure 4). The marked reduction in ERK phosphorylation by the lowest concentration of trametinib (5 nM) in the absence of a growth inhibitory effect suggests the presence of redundant pathways controlling entry into the cell division cycle, but these may be cell line specific. Higher cellular ERK protein expression has been correlated with shorter survival in triple-negative breast cancer [35]. It was of interest that many, but not all, of the MCF-7 hormone resistant sub-lines were highly sensitive to trametinib, regardless of their estrogen receptor status, and that all of the triple negative sub-lines were sensitive, despite low levels of ERK phosphorylation (Figure 8). This low phosphorylation level may nevertheless be sufficient to sustain the signaling cascade and promote cell proliferation.

The second aim of our study was to examine growth inhibitory effects of combinations of inhibitors. The PI3K/AKT/mTOR and MEK/ERK pathways are interconnected by multiple points of convergence, cross-talk, and feedback [36]; inhibition of one pathway may affect signaling, positively or negatively, in the reciprocal pathway (Figure 1). Since MAPK (RAS-RAF-MEK-ERK) pathway deregulation was identified as a dominant determinant in cancer resistance to PI3K inhibitors [37], concurrent targeting of both pathways can be hypothesized to improve the efficacy of treatment and provide better clinical outcome [38]. Such strategies are currently under clinical investigation (NCT01390818 and NCT01347866). Here, we used cell line models to study combinations of agents that target both the PI3K/mTOR and MEK pathways. Combination of trametinib with everolimus in MCF-7, T47D and SKBr3 cells generally showed synergistic effects on growth, while combination of trametinib with the mTOR/PI3K dual inhibitor NVP-BEZ235 showed weak antagonistic effects in T47D cells at some concentrations tested (Table 1, Figure 6). Dual inhibitors may inhibit mTOR directly, or activate it indirectly via stimulation of BRAF-MEK (Figure 1). Another study has reported that concurrent blockade of the PI3K/AKT/mTOR (with mTOR inhibitor AZD8055) and RAS/MEK/ERK (with MEK inhibitor AZD6244) pathways synergistically inhibits rhabdomyosarcoma cell growth [39]. A further study has shown that a combination of specific PI3K inhibitors (GDC-0941), rather than dual mTOR/PI3K inhibitors (NVP-BEZ235), with MEK inhibitors results in synergy in colorectal cancer cell lines [22]. Taken together, these results suggest the presence of cell line-dependent crosstalk among multiple pathways.

The signaling response of concurrent targeting of both PI3K/mTOR and MAPK pathways did not predict growth inhibitory effects in MCF-7, T47D and SKBr3 breast cancer cell lines. Everolimus efficiently suppressed phosphorylation of p70S6K and rpS6 in MCF-7, T47D and SKBr3 cell lines with and without trametinib treatment. AKT phosphorylation was down regulated by NVP-BEZ235 alone in the three cell lines, but the inhibitory effect was abrogated by the addition of trametinib in SKBr3 cells. GSK2126458 inhibited AKT phosphorylation in all cell lines tested, either alone or in combination with trametinib, but additive effects on growth were observed in SKBr3 when combined with trametinib. Therefore, the inactivation of AKT (PI3K pathway), with or without ERK activation, cannot predict growth sensitivity. Rapamycin and AKT inhibitor uniformly abrogated mTOR inhibition-induced AKT activation but failed to induce antitumor responses in a subset of head and neck cancers *ex vivo* [40].

In conclusion, the lack of efficacy of protein phosphorylation measurement as a predictor of trametinib sensitivity adds to the difficulty of identifying predictive biomarkers for targeted anticancer therapy. Furthermore, utilization of the MEK-ERK signaling pathways, as determined by phosphorylation of signaling components, varied widely across a series of cell lines and did not directly reflect mutation of genes coding these components [41]. Our study implies that examination of the degree of ERK phosphorylation in tumor biopsies will not predict for therapeutic response, and emphasizes the complexity and cell line specificity of cellular signaling pathways that govern response to individual targeted drugs.

## Supporting Information

Figure S1 **Drug combination effect on PARP in MCF-7, T47D and SKBr3 cell lines after 24 h as demonstrated by immunoblotting with anti-PARP antibodies.**
(EPS)

Figure S2 **Phosphorylation of p70S6K, AKT, and ERK in the breast cancer cell lines, including MCF-7 and its sub-lines (A, B, C and D).** Immunoblots with antibodies specific for phosphorylated and total protein are indicated below the corresponding control. Tubulin or actin are the loading control. Bands are normalized to tubulin or actin control and expressed as the mean from two experiments. The immunoblot for MCF-7 and its fulvestrant sub-lines were adapted from Leung et al. [28].
(TIF)

## References

[1] Montagut C, Settleman J (2009) Targeting the RAF-MEK-ERK pathway in cancer therapy. Cancer Lett 283: 125–134. 19217204 10.1016/j.canlet.2009.01.022

[2] Baselga J (2011) Targeting the phosphoinositide-3 (PI3) kinase pathway in breast cancer. Oncologist 16 Suppl 1 12–19. 10.1634/theoncologist.2011-S1-12 21278436

[3] Thomas RS, Sarwar N, Phoenix F, Coombes RC, Ali S (2008) Phosphorylation at serines 104 and 106 by Erk1/2 MAPK is important for estrogen receptor-alpha activity. J Mol Endocrinol 40: 173–184. 18372406 10.1677/JME-07-0165 PMC2277492

[4] Ghayad SE, Vendrell JA, Ben Larbi S, Dumontet C, Bieche I, et al (2010) Endocrine resistance associated with activated ErbB system in breast cancer cells is reversed by inhibiting MAPK or PI3K/Akt signaling pathways. Int J Cancer 126: 545–562. 19609946 10.1002/ijc.24750

[5] Gee JM, Robertson JF, Ellis IO, Nicholson RI (2001) Phosphorylation of ERK1/2 mitogen-activated protein kinase is associated with poor response to anti-hormonal therapy and decreased patient survival in clinical breast cancer. Int J Cancer 95: 247–254. 11400118 10.1002/1097-0215(20010720)95:4<247::aid-ijc1042>3.0.co;2-s

[6] Osborne CK, Schiff R (2011) Mechanisms of endocrine resistance in breast cancer. Annu Rev Med 62: 233–247. 20887199 10.1146/annurev-med-070909-182917 PMC3656649

[7] Adeyinka A, Nui Y, Cherlet T, Snell L, Watson PH, et al (2002) Activated mitogen-activated protein kinase expression during human breast tumorigenesis and breast cancer progression. Clin Cancer Res 8: 1747–1753. 12060612

[8] Rinehart J, Adjei AA, Lorusso PM, Waterhouse D, Hecht JR, et al (2004) Multicenter phase II study of the oral MEK inhibitor, CI-1040, in patients with advanced non-small-cell lung, breast, colon, and pancreatic cancer. J Clin Oncol 22: 4456–4462. 15483017 10.1200/JCO.2004.01.185

[9] O'Brien C, Wallin JJ, Sampath D, GuhaThakurta D, Savage H, et al (2010) Predictive biomarkers of sensitivity to the phosphatidylinositol 3' kinase inhibitor GDC-0941 in breast cancer preclinical models. Clin Cancer Res 16: 3670–3683. 20453058 10.1158/1078-0432.CCR-09-2828

[10] Nazarian RM, Prieto VG, Elder DE, Duncan LM (2010) Melanoma biomarker expression in melanocytic tumor progression: a tissue microarray study. J Cutan Pathol 37 Suppl 1 41–47. 20482674 10.1111/j.1600-0560.2010.01505.x

[11] Stones CJ, Kim JE, Joseph WR, Leung E, Marshall ES, et al (2013) Comparison of responses of human melanoma cell lines to MEK and BRAF inhibitors. Front Genet 4: 66. 23658559 10.3389/fgene.2013.00066 PMC3647113

[12] Kim KB, Kefford R, Pavlick AC, Infante JR, Ribas A, et al (2013) Phase II study of the MEK1/MEK2 inhibitor Trametinib in patients with metastatic BRAF-mutant cutaneous melanoma previously treated with or without a BRAF inhibitor. J Clin Oncol 31: 482–489. 23248257 10.1200/JCO.2012.43.5966 PMC4878037

[13] Jing J, Greshock J, Holbrook JD, Gilmartin A, Zhang X, et al (2012) Comprehensive predictive biomarker analysis for MEK inhibitor GSK1120212. Mol Cancer Ther 11: 720–729. 22169769 10.1158/1535-7163.MCT-11-0505

[14] Menzies AM, Long GV (2014) Dabrafenib and trametinib, alone and in combination for BRAF-mutant metastatic melanoma. Clin Cancer Res 20: 2035–2043. 24583796 10.1158/1078-0432.CCR-13-2054

[15] Gilmartin AG, Bleam MR, Groy A, Moss KG, Minthorn EA, et al (2011) GSK1120212 (JTP-74057) is an inhibitor of MEK activity and activation with favorable pharmacokinetic properties for sustained in vivo pathway inhibition. Clin Cancer Res 17: 989–1000. 21245089 10.1158/1078-0432.CCR-10-2200

[16] Baselga J, Campone M, Piccart M, Burris HA 3rd, Rugo HS, et al (2012) Everolimus in postmenopausal hormone-receptor-positive advanced breast cancer. N Engl J Med 366: 520–529. 22149876 10.1056/NEJMoa1109653 PMC5705195

[17] Knight SD, Adams ND, Burgess JL, Chaudhari AM, Darcy MG, et al (2010) Discovery of GSK2126458, a Highly Potent Inhibitor of PI3K and the Mammalian Target of Rapamycin. ACS Med Chem Lett 1: 39–43. 24900173 10.1021/ml900028r PMC4007793

[18] Maira SM, Stauffer F, Brueggen J, Furet P, Schnell C, et al (2008) Identification and characterization of NVP-BEZ235, a new orally available dual phosphatidylinositol 3-kinase/mammalian target of rapamycin inhibitor with potent in vivo antitumor activity. Mol Cancer Ther 7: 1851–1863. 18606717 10.1158/1535-7163.MCT-08-0017

[19] Serra V, Markman B, Scaltriti M, Eichhorn PJ, Valero V, et al (2008) NVP-BEZ235, a dual PI3K/mTOR inhibitor, prevents PI3K signaling and inhibits the growth of cancer cells with activating PI3K mutations. Cancer Res 68: 8022–8030. 18829560 10.1158/0008-5472.CAN-08-1385

[20] Hollestelle A, Elstrodt F, Nagel JH, Kallemeijn WW, Schutte M (2007) Phosphatidylinositol-3-OH kinase or RAS pathway mutations in human breast cancer cell lines. Mol Cancer Res 5: 195–201. 17314276 10.1158/1541-7786.MCR-06-0263

[21] Valentino JD, Li J, Zaytseva YY, Mustain WC, Elliott VA, et al (2014) Cotargeting the PI3K and RAS Pathways for the Treatment of Neuroendocrine Tumors. Clin Cancer Res 20: 1212–1222. 24443523 10.1158/1078-0432.CCR-13-1897 PMC3947505

[22] Haagensen EJ, Kyle S, Beale GS, Maxwell RJ, Newell DR (2012) The synergistic interaction of MEK and PI3K inhibitors is modulated by mTOR inhibition. Br J Cancer 106: 1386–1394. 22415236 10.1038/bjc.2012.70 PMC3326670

[23] Chou TC (2010) Drug combination studies and their synergy quantification using the Chou-Talalay method. Cancer Res 70: 440–446. 20068163 10.1158/0008-5472.CAN-09-1947

[24] Leung E, Kannan N, Krissansen GW, Findlay MP, Baguley BC (2010) MCF-7 breast cancer cells selected for tamoxifen resistance acquire new phenotypes differing in DNA content, phospho-HER2 and PAX2 expression, and rapamycin sensitivity. Cancer Biol Ther 9: 717–724. 20234184 10.4161/cbt.9.9.11432

[25] Whitehead RH, Monaghan P, Webber LM, Bertoncello I, Vitali AA (1983) A new human breast carcinoma cell line (PMC42) with stem cell characteristics. II. Characterization of cells growing as organoids. J Natl Cancer Inst 71: 1193–1203. 6606728

[26] Leung E, Kim JE, Rewcastle GW, Finlay GJ, Baguley BC (2011) Comparison of the effects of the PI3K/mTOR inhibitors NVP-BEZ235 and GSK2126458 on tamoxifen-resistant breast cancer cells. Cancer Biol Ther 11: 938–946. 21464613 10.4161/cbt.11.11.15527 PMC3127046

[27] Leung E, Rewcastle GW, Joseph WR, Rosengren RJ, Larsen L, et al (2012) Identification of cyclohexanone derivatives that act as catalytic inhibitors of topoisomerase I: effects on tamoxifen-resistant MCF-7 cancer cells. Invest New Drugs 30: 2103–2112. 22105790 10.1007/s10637-011-9768-4 PMC3484282

[28] Leung E, Kim JE, Askarian-Amiri M, Finlay GJ, Baguley BC (2014) Evidence for the Existence of Triple-Negative Variants in the MCF-7 Breast Cancer Cell Population. BioMed Res Int 2014: 7. 10.1155/2014/836769 PMC3960520 24724101

[29] Garcia-Echeverria C, Stauffer F, Furet P (2006) Preparation of imidazo[4,5-c]quinolin-2-ones and -thiones as lipid, PI3 and/or DNA protein kinase inhibitors with therapeutic uses. PCT Int Appl WO 2006122806 A2.

[30] Stowasser F, Baenziger M, Garad SD (2008) Preparation of salts and crystalline forms of 2-methyl-2-[4-(3-methyl-2-oxo-8-quinolin-3-yl-2,3-dihydroimidazo[4,5-c]quinolin-1-yl)-phenyl]propionitrile and its use as a drug. PCT Int Appl WO 2008064093A2.

[31] Barlund M, Monni O, Kononen J, Cornelison R, Torhorst J, et al (2000) Multiple genes at 17q23 undergo amplification and overexpression in breast cancer. Cancer Res 60: 5340–5344. 11034067

[32] Corcoran RB, Settleman J, Engelman JA (2011) Potential therapeutic strategies to overcome acquired resistance to BRAF or MEK inhibitors in BRAF mutant cancers. Oncotarget 2: 336–346. 21505228 10.18632/oncotarget.262 PMC3248170

[33] Aksamitiene E, Kholodenko BN, Kolch W, Hoek JB, Kiyatkin A (2010) PI3K/Akt-sensitive MEK-independent compensatory circuit of ERK activation in ER-positive PI3K-mutant T47D breast cancer cells. Cell Signal 22: 1369–1378. 20471474 10.1016/j.cellsig.2010.05.006 PMC2893265

[34] Yip-Schneider MT, Klein PJ, Wentz SC, Zeni A, Menze A, et al (2009) Resistance to mitogen-activated protein kinase kinase (MEK) inhibitors correlates with up-regulation of the MEK/extracellular signal-regulated kinase pathway in hepatocellular carcinoma cells. J Pharmacol Exp Ther 329: 1063–1070. 19258520 10.1124/jpet.108.147306

[35] Bartholomeusz C, Gonzalez-Angulo AM, Liu P, Hayashi N, Lluch A, et al (2012) High ERK protein expression levels correlate with shorter survival in triple-negative breast cancer patients. Oncologist 17: 766–774. 22584435 10.1634/theoncologist.2011-0377 PMC3380875

[36] Saini KS, Loi S, de Azambuja E, Metzger-Filho O, Saini ML, et al (2013) Targeting the PI3K/AKT/mTOR and Raf/MEK/ERK pathways in the treatment of breast cancer. Cancer Treat Rev 39: 935–946. 23643661 10.1016/j.ctrv.2013.03.009

[37] Yu K, Toral-Barza L, Shi C, Zhang WG, Zask A (2008) Response and determinants of cancer cell susceptibility to PI3K inhibitors: combined targeting of PI3K and Mek1 as an effective anticancer strategy. Cancer Biol Ther 7: 307–315. 18059185 10.4161/cbt.7.2.5334

[38] Fruman DA, Rommel C (2014) PI3K and cancer: lessons, challenges and opportunities. Nat Rev Drug Discov 13: 140–156. 24481312 10.1038/nrd4204 PMC3994981

[39] Larsen KB, Lamark T, Overvatn A, Harneshaug I, Johansen T, et al (2010) A reporter cell system to monitor autophagy based on p62/SQSTM1. Autophagy 6: 784–793. 20574168 10.4161/auto.6.6.12510

[40] Radhakrishnan P, Baraneedharan U, Veluchamy S, Dhandapani M, Pinto DD, et al (2013) Inhibition of rapamycin-induced AKT activation elicits differential antitumor response in head and neck cancers. Cancer Res 73: 1118–1127. 23361299 10.1158/0008-5472.CAN-12-2545

[41] Kim JE, Stones C, Joseph WR, Leung E, Finlay GJ, et al (2012) Comparison of growth factor signalling pathway utilisation in cultured normal melanocytes and melanoma cell lines. BMC Cancer 12: 141. 22475322 10.1186/1471-2407-12-141 PMC3352269

[42] Kuntzer J, Maisel D, Lenhof HP, Klostermann S, Burtscher H (2011) The Roche Cancer Genome Database 2.0. BMC Med Genomics 4: 43. 21586118 10.1186/1755-8794-4-43 PMC3114700

