# Supplementary figures and images for: Relationships between Signaling Pathway Usage and Sensitivity to a Pathway Inhibitor: Examination of Trametinib Responses in Cultured Breast Cancer Lines
Source: PLoS One. 2014 Aug 29;9(8):e105792. doi: 10.1371/journal.pone.0105792 (PMC4149495; doi:10.1371/journal.pone.0105792)

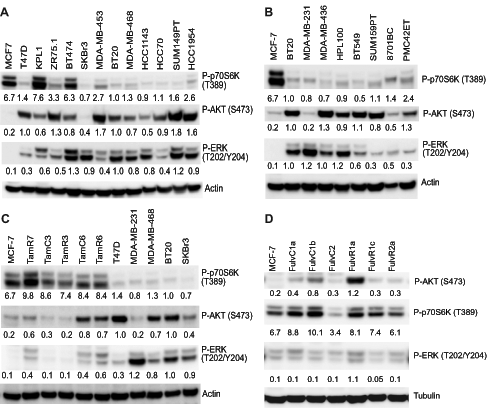

Supplement: Figure S2 — Phosphorylation of p70S6K, AKT, and ERK in the breast cancer cell lines, including MCF-7 and its sub-lines (A, B, C and D). Immunoblots with antibodies specific for phosphorylated and total protein are indicated below the corresponding control. Tubulin or actin are the loading control. Bands are normalized to tubulin or actin control and expressed as the mean from two experiments. The immunoblot for MCF-7 and its fulvestrant sub-lines were adapted from Leung et al. [28]. (TIF) [file pone.0105792.s002.tif]
